# Developing an entrustable professional activity for providing health education and consultation in occupational therapy and examining its validity

**DOI:** 10.1186/s12909-024-05670-1

**Published:** 2024-06-28

**Authors:** Chung-Pei Fu, Ching-Kai Huang, Yi-Chiun Yang, Wei-Sheng Liao, Shih-Min Huang, Wei-Di Chang, Yi-Ju Chen, Ming-Wei Li, Yi-Ju Lin, Chin-Lung Wu, Hsin-Yu Chi, Chia-Yi Lee, Fu-Mei Chiang, Yu-Lan Chen, Ching-Fen Tsou, Tzu-Hung Liu, Chia-Ting Su, Ai-Lun Yang, Nung-Chen Kuo, Wan-Ying Chang

**Affiliations:** 1 Department of Occupational Therapy, College of Medicine, Fu Jen Catholic University, New Taipei City, Taiwan; 2 Department of Rehabilitation, Occupational Therapy, Linkuo Chang Gung Memorial Hospital, Taoyuan, Taiwan; 3 Department of Rehabilitation, Lotung Poh-Ai Hospital, Yilan, Taiwan; 4 Department of Rehabilitation, Taipei Medical University Hospital, Taipei, Taiwan; 5 Department of Rehabilitation, Sijhih Cathay General Hospital, New Taipei City, Taiwan; 6 Department of Rehabilitation, Mackay Memorial Hospital, Taipei, Taiwan; 7 Department of Rehabilitation, Kaohsiung Chang Gung Memorial Hospital, Kaohsiung, Taiwan; 8 Department of Rehabilitation, Changhua Christian Hospital, Changhua, Taiwan; 9 Department of Rehabilitation, Cathay General Hospital, Taipei, Taiwan; 10 Department of Physical Medicine and Rehabilitation, National Taiwan University Hospital, Taipei, Taiwan; 11 Department of Physical Medicine and Rehabilitation, Taipei Veterans General Hospital, Taipei, Taiwan; 12 Department of Family Medicine, Taipei Tzu Chi Hospital, New Taipei City, Taiwan; 13 School of Medicine, Tzu Chi University, Hualien City, Taiwan; 14 Institute of Sports Sciences, University of Taipei, Taipei, Taiwan; 15 Department of Occupational Therapy, Taoyuan General Hospital, Ministry of Health and Welfare,, No. 1492, Zhongshan Rd.,Taoyuan Dist., Taoyuan, 330 Taiwan; 16 Department of Physical Medicine and Rehabilitation, Taipei Hospital, Ministry of Health and Welfare, No. 127, Su-Yuan Rd., Hsin-Chung Dist., New Taipei City, Taiwan

**Keywords:** Entrustable Professional Activity, Occupational therapy education, Competency-based medical education

## Abstract

**Background:**

Entrustable Professional Activities (EPA)-based assessment is easily and intuitively used in evaluating the learning outcomes of competency-based medical education (CBME). This study aimed to develop an EPA for occupational therapy focused on providing health education and consultation (TP-EPA3) and examine its validity.

**Methods:**

Nineteen occupational therapists who had completed online training on the EQual rubric evaluation participated in this study. An expert committee identified six core EPAs for pediatric occupational therapy. TP-EPA3 was developed following the EPA template and refined through consensus meetings. The EQual rubric, a 14-item, five-point criterion-based anchor system, encompassing discrete units of work (DU), entrustable, essential, and important tasks of the profession (EEIT), and curricular role (CR), was used to evaluate the quality of TP-EPA3. Overall scores below 4.07, or scores for DU, EEIT, and CR domains below 4.17. 4.00, and 4.00, respectively, indicate the need for modifications.

**Results:**

The TP-EPA3 demonstrated good validity, surpassing the required cut-off score with an average overall EQual score of 4.21 (*SD* = 0.41). Specific domain scores for DU, EEIT, and CR were 3.90 (*SD* = 0.69), 4.46 (*SD* = 0.44), and 4.42 (*SD* = 0.45), respectively. Subsequent revisions clarified observation contexts, enhancing specificity and focus. Further validation of the revised TP-EPA3 and a thorough examination of its reliability and validity are needed.

**Conclusion:**

The successful validation of TP-EPA3 suggests its potential as a valid assessment tool in occupational therapy education, offering a structured approach for developing competency in providing health education and consultation. This process model for EPA development and validation can guide occupational therapists in creating tailored EPAs for diverse specialties and settings.

**Supplementary Information:**

The online version contains supplementary material available at 10.1186/s12909-024-05670-1.

## Introduction

Competency-based medical education (CBME) represents a strategic evolution in the methodology of medical training, emphasizing the development of specific competencies essential for effective clinical practice [1, 2]. Although competency frameworks have been developed by the Accreditation Council for Graduate Medical Education (ACGME) and Canadian Medical Education Directives for Specialists (CanMEDS), their implementation in clinical practice has been limited. Entrustable professional activities (EPAs) and milestones have emerged to bridge the gap between competency frameworks and assessment and training in clinical practices [3–5].

EPAs are discrete clinical activities that require the integration of various competencies and represent an activity associated with a specific clinical event [6, 7], whereas milestones refer to observable markers along a continuum of progress [8]. However, the application of milestones as evaluative tools for trainees’ competency faces challenges due to their sheer number, the extensive and complex training required, and the necessity for full participation in the learning process [9]. Therefore, EPA-based assessment is easier and more intuitive to use in evaluating the learning outcomes of the CBME [10, 11].

EPAs have been well developed in many medical professions, such as medicine of various specialties [12–18], dentistry [19], nursing [20], pharmacy [21, 22], radiology [23–25], physical therapy [26], and occupational therapy [27, 28]. While EPAs tailored to occupational therapy have been established in Singapore, they primarily focus on undergraduate education during the earlier years of study, emphasizing fundamental professional activities crucial for early-stage clinical education. However, it is crucial to develop EPAs specific to the final year of undergraduate clinical training and post-graduate clinical training. During this phase, occupational therapy students are exposed to four major domains: physical, mental, pediatric, and community. Each domain encompasses unique core competencies and professional activities. Thus, developing EPAs tailored to each domain of occupational therapy is essential. Accordingly, the purpose of this study was to delineate the process of developing an EPA in pediatric occupational therapy, using the EPA3 Providing Health Education and Consultation in occupational therapy (TP-EPA3) serving as an example, and to examine its validity. While we developed six EPAs in pediatric occupational therapy, this study focuses exclusively on TP-EPA3 as a representative example due to its broad applicability across the four major domains of occupational therapy: physical, mental, pediatric, and community.

## Methods

This study was approved by the institutional review boards of Fu Jen Catholic University (C110093) and Taipei Hospital, Ministry of Health and Welfare (TH-IRB-0022–0027).

### Participants

Nineteen occupational therapists (11 females, 8 males), who had completed online training on the EQual rubric evaluation, rated the 6 core EPAs in pediatric occupational therapy on the EQual rubric. Their age distribution was as follows: 21.1% were aged 31–40 years; 63.2%, 41–50 years; and 15.8%, 51–60 years. Their workplaces were medical centers (42.1%), regional teaching hospitals (47.4%), and district teaching hospitals (5.3%). Their positions were chiefs of occupational therapy (26.3%), chiefs of pediatric occupational therapy (15.8%), teaching directors of occupational therapy (26.3%), clinical teachers (57.9%), and university teachers. The average duration of their work experience was 20.2 years (*SD* = 6.7), whereas that as clinical teachers was 15.6 years (*SD* = 7.8).

### Procedure

This study comprised two stages: the development of TP-EPA3, and an examination of the structure and quality of TP-EPA3.

In the first stage, six topics for the core EPAs were identified by expert committee using the nominal group technique and survey questionnaires distributed to 131 teaching hospitals in Taiwan [29]. The expert committee included two university teachers from departments of occupational therapy, 24 clinical teachers of pediatric occupational therapy in teaching hospitals, and one external expert developing EPAs in family medicine in Taiwan. The nominal group technique and survey questionnaires were chosen to ensure a comprehensive and systematic collection of expert opinions and have been detailed in previous studies [30, 31]. The survey was distributed to 131 teaching hospitals in Taiwan, providing a broad basis for the identification of core EPAs [29].

The Taiwan Occupational Therapy Association made only minor textual refinements to the titles of the six core EPAs: EPA1, “Providing evaluations in occupational therapy”; EPA2, “Providing interventions in occupational therapy”; EPA3, “Providing health education and consultation” (TP-EPA3); EPA4, “Writing occupational therapy medical records”; EPA5, “Providing transdisciplinary collaboration healthcare”; and EPA6, “Providing services of splints or assistive devices”, without altering the core competencies or the content of the EPAs themselves. The draft of TP-EPA3 was written by two pediatric occupational therapy clinical teachers (corresponding and co-corresponding authors) from two teaching hospitals within the expert committee, based on the EPA template [11]. Following three rounds of consensus meetings, the expert committee finalized the description of TP-EPA3, as shown in Appendix 1.

In the second stage, 16 committee members from the stage one expert committee, along with 3 non-committee occupational therapy experts with EPA experience, rated TP-EPA3. All raters had completed the online training of the EQual rubric evaluation and assessed TP-EPA3 according to the EQual rubric.

### Measure

The EQual rubric is a 14-item evaluation utilized to assess the quality of EPAs [32]. This rubric measures the constructs of EPAs across 3 domains, including discrete units of work (DU) (items 1–6), entrustable, essential, and important tasks of the profession (EEIT)(items 7–10), and curricular role (CR) (items 11–14) [32]. Each item is scored using a five-point criterion-based anchor system [32], and an online training video is available for scoring [33]. A cut-off score of 4.07 determines whether a given EPA requires modification, with an average overall EQual score below 4.07 indicating that it does [32]. Moreover, the cutoff scores for the DU, EEIT, and CR domains are 4.17, 4.00, and 4.00, respectively [34]. The EQual rubric evaluation has been found to be reliable, valid and useful in EPA development [32, 34, 35].

### Data analysis

Data analyses were performed in Microsoft Excel 16.75 for Mac. We calculated mean and standard deviation for the EQual rubric score and three domain scores to determine whether the EPA needs modification. The EQual rubric score represented the average of all 14 items, and domain scores for DU, EEIT, and CR were calculated from items 1–6, 7–10, and 11–14, respectively [32, 34, 35]. Additionally, the scatter plot was used to examine the dispersion of scores across the three domains.

## Results

### Development of EPA3, providing health education and consultation (TP-EPA3)

The description of TP-EPA3 is shown in Appendix 1. The EPA comprises 8 parts: title; specifications and limitations; potential risks in case of failure; most relevant competency domains; required knowledge, skills, attitude and experiences; assessment information sources to assess progress and ground a summative entrustment decision; entrustment for which level of supervision is to be reached at which stage of training; and expiration date.

The entrustment and supervision scale for TP-EPA3 adopted Chen’s entrustment and supervision scale [36]. Specifically, clinical teachers were asked to assess the trainee's level of entrustment using the following question: "If you were to supervise this trainee again in a similar professional task and situation, which of the following statements aligns with how you would assign the task?" This question was used to guide the clinical teachers' entrustment decisions. The entrustment and supervision scale comprised 5 levels, with level 1 and level 2 being further divided into two sublevels, and level 3 being divided into three sublevels (Appendix 2). The definition of level 1a was “Not allowed to observe practicing the EPA”. Level 1b was “Not allowed to practice the EPA; allowed to observe”. Level 2a was "Allowed to practice the EPA only under proactive, full supervision as co-activity with supervisor”. Level 2b was “Allowed to practice the EPA only under proactive, full supervision with supervision in room ready to step in as needed”. Level 3a was “Allowed to practice the EPA only under reactive/on-demand supervision with supervisor immediately available, all findings double-checked”. Level 3b was “Allowed to practice the EPA only under reactive/on-demand supervision with supervision immediately available, key findings double-checked”. Level 3c was “Allowed to practice the EPA only under reactive/on-demand supervision with supervisor distantly available, findings reviewed”. Level 4 was “Allowed to practice the EPA unsupervised”. Level 5 was “Allowed to supervise others in practice of the EPA”.

### Results of the EQual rubric

**Fig. 1:**
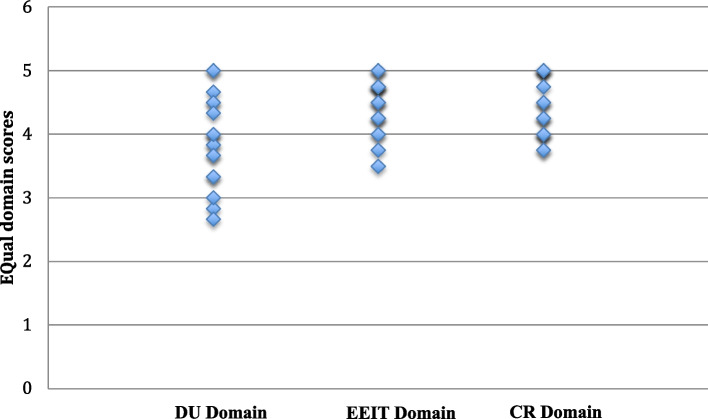
Scatter plot of the three EQual domain scores of TP-EPA3: Providing Health Education and Consultation in pediatric occupational therapy in Taiwan. Note: EPA = entrustable professional activity. DU = Discrete units of work. EEIR = Entrustable, essential, and important tasks of the profession. CR = Curricular role

Eighteen occupational therapists rated the TP-EPA3 according to the EQual rubric. The response rate was 94.7%. The EQual item, domain, and overall scores for the TP-EPA3 are listed in Table 1. The average scores of the individual items on the Equal rubric ranged from 3.17 to 4.83. The average overall EQual score was 4.21 (*SD* = 0.41), which was higher than the cut-off score, 4.07. The domain scores for DU, EEIT, and CR were 3.90 (*SD* = 0.69), 4.46 (*SD* = 0.44), and 4.42 (*SD* = 0.45), respectively. Only the domain score for DU (3.90) was lower than the domain cut-off score (4.17). The scatter plot of the three domain scores of the EQual is shown in Fig. 1.

**Table 1:** EQual rubric item, domain, and overall scores for the TP-EPA3

	Item	Mean (SD)	Median	Mode
1	This EPA has a clearly defined beginning and end	3.17 (1.42)	3	3,4,5
2	This EPA is independently executable to achieve a defined clinical outcome	4.06 (1.0)	4	4
3	This EPA is specific and focused	3.94 (0.94)	4	4,5
4	This EPA is observable in process	4.17 (0.51)	4	4
5	This EPA is measurable in outcome	4.05 (0.64)	4	4
6	This EPA is clearly distinguished from other EPAs in the framework	4.00 (0.69)	4	4
7	This EPA describes work that is essential and important to the profession	4.72 (0.46)	5	5
8	Performing this EPA leads to recognized output or outcome of labor	4.28 (0.83)	4.5	5
9	The performance of this EPA in clinical practice is restricted to qualified personnel	4.00 (0.97)	4	5
10	This EPA addresses professional work that is suitable for entrustment	4.83 (0.38)	5	5
11	This EPA requires the application of knowledge, skills, and/or attitudes (KSAs) acquired through training	4.39 (0.50)	4	4
12	This EPA involves application and integration of multiple domains of competence	4.67 (0.49)	5	5
13	The EPA title describes a task, not qualities or competencies of a learner	4.33 (0.49)	4	4
14	This EPA describes a task and avoids adjectives (or adverbs) that refer to proficiency	4.28 (0.75)	4	5
Domain 1: Discrete units of work (average score of Item 1 to item 6) (cut-off score: 4.17)		**3.90 (0.69)**		
Domain 2: Entrustable, essential, and important tasks of the profession (average score of item 7 to item 10) (cut-off score: 4.00)		4.46 (0.44)		
Domain 3: Curricular role (average score of item 11 to item 14) (cut-off score: 4.00)		4.42 (0.45)		
Average overall EQual score (cut-off score: 4.07)		4.21 (0.50)		

*EPA* Entrustable Professional Activity

## Discussion

To the best of our knowledge, this study was the first to illustrate the development process of EPAs in pediatric occupational therapy, specifically tailored for use in the final year of undergraduate clinical training and post-graduate clinical training in the pediatric domain. The EPAs developed in this study can complement those developed in Singapore [27, 28], which are utilized during the earlier years of undergraduate education. Early exposure to EPAs in undergraduate education can enhance students' understanding of the EPA concept, increase clinical engagement, foster a stronger sense of professional identity, bridge the gap between theoretical knowledge and clinical practice, and facilitate comprehension of future practice expectations during conditional registration [28, 37, 38]. Using EPAs in pediatric occupational therapy during the final year of undergraduate clinical training and post-graduate clinical training can assess students' advanced clinical skills in pediatric occupational therapy domain, enable students to engage in self-directed learning to address their weakness, as well as determine their readiness to become independent pediatric occupational therapists [39, 40].

The overall EQual score of TP-EPA3 (4.21), being higher than the cut-off score of 4.07, indicated good validity and quality. However, the EQual domain score for DU (3.90), below the cut-off score of 4.17, indicated a need for further revisions of the items within this domain. The scatter plot revealed greater variability in EQual domain scores for DU than for the other domains. Notably, the average scores for item 1 (“This EPA has a clearly defined beginning and end”) and item 3 (“This EPA is specific and focused”) were the lowest (Table 1). In pediatric occupational therapy, health education and consultations frequently occurred during various interactions with parents, such as following screenings, assessments, interventions, cessation of interventions, or even during casual conversations about the child's recent behaviors or challenges. Therapists often used these opportunities to recommend home program, activity adjustments, environmental modifications, or changes in parenting strategies. Due to the nature of these interactions, it would be challenging to identify a clear beginning and end for providing consultations and health education. This might explain why items 1 and 3 received lower scores. Consequently, the observation contexts of TP-EPA3 were refined to focus on four specific contexts: after screening, after evaluation, after intervention, and intervention discontinuation. These specifications were revised in alignment with the definitions, sequences, and crucial observation points related to providing health education and consultation. The revised version of the TP-EPA3 is provided in Appendix 3.

The TP-EPA3 presented in this paper may not be universally applicable to all occupational therapy fields, such as physical, mental, and community settings, or at different levels of hospitals or community agencies, or in all countries. However, occupational therapists can employ the EPA topic development process [29] and EPA content development process in this paper to develop their core EPAs tailored to their respective fields, hospitals or community agencies, or countries. In cases where occupational therapy units provide specialized services or interventions, specialized EPAs may also be developed by following this EPA development process. For instance, if an occupational therapy unit specializes in telemedicine or screening, specialized EPAs can be developed. Moreover, if only certain components of the professional tasks of the EPAs can be performed due to the size or other constraints, nested EPAs [11], which are smaller units of the original EPA, can be considered.

The assessment information sources utilized in evaluating progress and grounding a summative entrustment decision for the TP-EPA3, as presented in this study, encompass all the assessment methods typically employed to evaluate trainees’ capabilities. Occupational therapy clinical teachers can select the assessment methods they already utilize from those provided in the TP-EPA3. The key point in determining the summative entrustment and supervision levels for EPAs is to thoroughly consider the outcomes of multiple assessment methods and assessments. This approach prevents trainees from being unfairly labeled as “under proactive” based solely on one performance or poor performance on severe patients [41].

Since our EPAs were designed to evaluate both UGY and PGY trainees, Chen’s prospective entrustment and supervision scale [36] was adopted for two main reasons. First, the prospective nature of Chen’s scale could reduce the influence of contextual factors such as time of observation and work load, as well as task factors such as complexity of patient’s conditions [41]. Second, Chen’s scale expands the lower levels of the scale to include finer gradation of supervision, making it more suitable for assessing the performances of UGY trainees [36]. Thus, the scale offers a more comprehensive framework for evaluating the trainees’ performances at different stages of training, allowing more detailed analysis of their progress.

### Limitations and suggestions

This study had four major limitations. First, although the overall EQual score of the initial TP-EPA3 indicated acceptable content validity, the revised TP-EPA3 still needs to be examined to determine whether the DU domain score has been improved. Second, since only the content validity of TP-EPA3 was examined, its inter-rater and intra-rater reliability, convergent and discriminant validity, and responsiveness should be further investigated. Third, the target entrustment and supervision levels of UGY and PGY trainees were recommended by the expert committee. Future studies should investigate the perspectives of occupational therapy clinical teachers on these target levels. Fourth, a potential limitation of our study is the overlap of committee members across the development and evaluation phases of TP-EPA3. Sixteen of the nineteen experts who assessed TP-EPA3 using the EQual rubric evaluation were involved in the initial development. Despite all raters being trained using the EQual rubric, their prior involvement could introduce unintentional rater bias, potentially influencing the impartiality of their assessments.

## Conclusion

EPAs provide a time-efficient, feedback-oriented, and workplace-based assessment for evaluating whether trainees can perform clinical professional tasks competently without supervision. The EPA development process outlined in this study can help occupational therapists develop core EPAs, specialized EPAs, or nested EPAs tailored to their specific fields, hospitals or community agencies, or countries. In conclusion, EPAs are not only easy and intuitive for assessing the learning outcomes of CBME but also support clinical teachers in evaluating trainee independence in significant clinical professional activities and in curriculum design. Furthermore, EPAs help trainees prepare for clinical professional activities. Specifically, the TP-EPA3, which focus on providing health education and consultation in pediatric occupational therapy in Taiwan, has been validated using the EQual rubric, paving the way for the development of additional core EPAs using the process described.

### Supplementary Information

Supplementary Material 1.

Supplementary Material 2.

Supplementary Material 3.

## Data Availability

Data is available upon reasonable request from corresponding author. The e-mail of the corresponding author is yves7116@mail.tygh.gov.tw and wanying0928@yahoo.com.tw .

## Abbreviations

EPA: Entrustable Professional Activity
CBME: Competency-based medical education
TP-EPA3: EPA3 for occupational therapy focused on providing health education and consultation
DU: Discrete units of work
EEIT: Entrustable, essential, and important tasks of the profession
CR: Curricular role
ACGME: Accreditation Council for Graduate Medical Education
CanMEDS: Canadian Medical Education Directives for Specialists
UGY: Undergraduate year
PGY: Post-graduate year
