# Supplementary material for: Developing an entrustable professional activity for providing health education and consultation in occupational therapy and examining its validity
Source: BMC Med Educ. 2024 Jun 28;24:705. doi: 10.1186/s12909-024-05670-1 (PMC11214254; doi:10.1186/s12909-024-05670-1)
Supplement: Supplementary file 2 — Supplementary Material 2. [file 12909_2024_5670_MOESM2_ESM.pdf]

Appendix 2 Entrustment and supervision scale used for EPAs in pediatric occupational therapy

Level 1	Level 2	Level 3	Level 4	Level 5
Not allowed to practice the EPA<br><br>1a Not allowed to observe<br>1b Allowed to observe only	Allowed to practice the EPA only under proactive, full supervision<br><br>2a As co-activity with supervisor<br>2b With supervisor in room ready to step in as needed	Allowed to practice the EPA only under reactive/on-demand supervision<br><br>3a With supervisor immediately available, all findings double-checked<br>3b With supervisor immediately available, key findings double-checked<br>3c With supervisor distantly available, findings reviewed	Allowed to practice the EPA unsupervised	Allowed to supervise others in practice of the EPA
