# Supplementary material for: Developing an entrustable professional activity for providing health education and consultation in occupational therapy and examining its validity
Source: BMC Med Educ. 2024 Jun 28;24:705. doi: 10.1186/s12909-024-05670-1 (PMC11214254; doi:10.1186/s12909-024-05670-1)
Supplement: Supplementary file 3 — Supplementary Material 3. [file 12909_2024_5670_MOESM3_ESM.pdf]

Appendix 3 Revised Entrustable Professional Activities in Pediatric Occupational Therapy

TP-EPA3: Providing Health Education and Consultation

<table>
<tr><td colspan="2">1. EPA Title: Providing health education and consultation</td></tr>
<tr><td colspan="2">2. Specifications and limitations</td></tr>
<tr><td rowspan="2">Provide occupational therapy health education and consultation for outpatients and inpatients in the contexts of screening, evaluation, intervention, and intervention discontinuation.
(1) Maintain a positive therapist–client relationship before health education and consultation, such as giving a brief self-introduction, communicating with clients and their family members, getting consent, protecting clients' confidentiality and privacy, and respecting for clients and their family members.
(2) Interview with the client-centered or family-centered approach. Listen and have empathy, understand their perspectives and feelings, and provide emotional support when receiving information from clients and their family members.
(3) Gather opinions continuously and exchange information and opinions.
(4) Recapitulate the content of the health education and consultation to make the issues clearly after summarizing.
(5) Clarify the core issues, implement knowledge translation, use communication skills, and communicate and explain with clients and their family members in a way they can understand.
(6) Apply occupational therapy knowledge to clinical reasoning and explain to the clients and their family members, and develop occupational therapy intervention strategies collaboratively.
(7) Provide occupational therapy intervention strategies that are appropriate, specific, quantified, and clearly executable home programs.
(8) Provide medical/ social resources and education related information as needed before the end of health education and consultation.</td><td>Limitations:
Refer to other relevant professionals in the conditions of non-occupational therapy domains with doubts about the appropriateness of applying occupational therapy, such as in cases of suspected child abuse or clients' companions not being their primary caregivers.</td></tr>
<tr><td>Context and targeted transition
Trainees must (but not limited to) be observed in the following contexts (at least one context) for the completion of training:
(1) After screening
(2) After evaluation
(3) After intervention
(4) Intervention discontinuation</td></tr>
<tr><td colspan="2">3. Potential risks in case of failure</td></tr>
</table>

(1) Unnecessary waste of medical resources
(2) Pressure on medical disputes and litigation
(3) Inappropriate emotional transference
(4) Negative effects on medical institutions and professionals, such as complaints, negative emotions, negative comments, etc.

4. Most relevant competency domains

(1) Values, knowledge, and skills
(2) Therapeutic and professional relationships
(3) Professional reasoning

5. Required knowledge, skills, attitude and experiences

**Knowledge:**	**Skill, attitudes:**	**Experience:**
(1) Knowledge of therapist–client communication and narrative medicine<br>(2) Complete the initial level of empathy (able to listen to and give empathetic feedback to the feelings and behaviors expressed by clients or their family members) and therapist–client communication training or courses<br>(3) Knowledge of resources related to social administration system, health administration system, and school system	(1) Communication and interview skills<br>(2) Skills of clinical reasoning and using frame of reference<br>(3) Abilities to develop teaching materials for health education	No experience required.

6. Assessment information sources to assess progress and ground a summative entrustment decision

Use multiple-methods and multiple-assessments to evaluate trainees' abilities, to enhance the validity of the EPAs in clinical practices, and to improve the reliability of the summative assessment:

(1) Knowledge test: Design a valid written test for the connotation of the tasks to confirm that the trainee has prior knowledge of the implementation of the treatment. The questions of the written test should focus on comprehension, analysis, judgment, and application to improve the validity of the evaluation.
(2) Case-based discussion (CbD): Test the clinical thinking, professional reasoning, and judgment abilities related to the EPA. The recommended evaluation tool is CbD.
(3) Short-practice observation: Observe and evaluate the actual performance of the trainee in clinical practice. The recommended evaluation tools include directly observed procedural skills (DOPS), mini-clinical evaluation exercise (mini-CEX), and ad-hoc EPA-based assessment.

(4)	Learning portfolio: Record the learning process, including quantitative (such as number of clients, and number of operations) and qualitative (such as self-evaluation, thoughts, and reflections) content, which can be used as a reference for accumulation of the learning experiences and demonstration of self-learning abilities. The recommended evaluation tools include case logs and case-reports.
(5)	Other activities that can be used to evaluate the trainees' entrustment level of their core competency.
**7.**	**Entrustment for which level of supervision is to be reached at which stage of training?**
(1)	The under-graduate year (UGY) trainees should reach Level 3c before the end of UGY training
(2)	The post-graduate year (PGY) trainees should reach Level 4 before the end of their PGY training.
(3)	Senior new employees should reach Level 4 after working for a half year and Level 5 after working for one year.
**8.**	**Expiration date**
The entrustment/supervision level should be re-evaluated and determined when an event (disease or accident) which may impair the trainee's abilities to perform medical treatment occurs, or when the training is interrupted for more than one year.	
